# Pilot Case Series of Robotic-Assisted Hernia Repair in Patients With Cirrhosis

**DOI:** 10.3389/jaws.2025.14226

**Published:** 2025-04-24

**Authors:** Dominic Amara, Faiz Shaik, Rhiannon Olivarez-Kidwell, Matthew Lin, Ian Soriano, Garrett Roll, Shareef Syed

**Affiliations:** ^1^ Department of Surgery, University of California, San Francisco, San Francisco, CA, United States; ^2^ Dumont-UCLA Transplant and Liver Cancer Centers, Department of Surgery, David Geffen School of Medicine at University of California, Los Angeles, Los Angeles, CA, United States

**Keywords:** robotic abdominal wall repair, liver transplant, ventral hernia repair, cirrhosis & portal hypertension, ascites

## Abstract

Umbilical and ventral hernias in patients with cirrhosis cause significant morbidity including flood syndrome, bowel obstruction, and pain and limit quality of life. Ascites and portal hypertension increase the risk of complications, resulting in apprehension with intervention and costly cycles of readmission. No studies have explored the safety or efficacy of robotic-assisted repair of primary umbilical hernias in this population. We performed a retrospective review of patients with cirrhosis at a single institution who underwent elective or emergent robotic hernia repair between June 2023 and May 2024. A total of 7 patients were included with a median MELD-Na of 17 (IQR 14–22) and the majority of whom (6 of 7, 85.7%) had ascites at the time of surgery. Three patients required emergent or urgent operations. No drains were required at the time of surgery. There were no Clavien-Dindo grade 3 or higher complications, no patients had leakage of ascites from their incisions, and no patients developed hernia recurrence (median follow-up 173 days). There were 2 Clavien-Dindo grade 1 or 2 complications: one superficial skin infection treated with antibiotics and one case of urinary retention. This limited series suggests that robotic hernia repair is technically feasible and safe in a select group of patients with cirrhosis including those with ascites. We propose an approach to robotic-assisted hernia repair in these complex patients.

## Introduction

The prevalence of hernias in patients with cirrhosis can be as high as 40% [1]. Umbilical and incisional hernias in patients with cirrhosis, particularly with ascites, cause significant morbidity including flood syndrome (uncontrolled leakage of ascites through a wound), small bowel obstruction, pain, malnutrition and liver decompensation [2]. Ascites and portal hypertension increase the risk of complications, resulting in fear about surgical repair. For patients with ascites, recommendations advocate for control of ascites followed by repair in ideal circumstances [3, 4]. However, this is not feasible for those with refractory ascites, or in the setting of bowel incarceration. For those with refractory ascites who are expected to undergo liver transplantation within 3–6 months, repair during or following transplantation is the preferred approach. If transplantation is not likely, drainage of ascites or a transjugular intrahepatic portosystemic shunt (TIPS) prior to repair are options [3, 5]. However, there are numerous patients for whom drainage or TIPS are not safe options, for example, those with hepatic encephalopathy, congestive heart failure or pulmonary artery hypertension. For these patients, conservative management is recommended [3]. However, this approach often remains perilous and commonly leads to costly readmissions for hernia-related issues (pain, obstruction), decompensation and even death [6]. When repair is attempted in these patients, it is often done out of necessity with an open approach and a significant risk of complications. As such, there is a need for novel surgical approaches to address hernias in patients with cirrhosis with refractory ascites. We present the first case series of robotic-assisted ventral hernia repair in patients with cirrhosis, the majority of whom had ascites, to assess whether it may be a safe and viable option in this population.

## Methods

### Patient Population

We performed a retrospective review of patients with cirrhosis at a single institution who underwent robotic umbilical or ventral incisional hernia repair between 6/2023 and 5/2024. This study was approved by our institutional review board (IRB 20-31396).

### Surgical Technique

**FIGURE 1:**
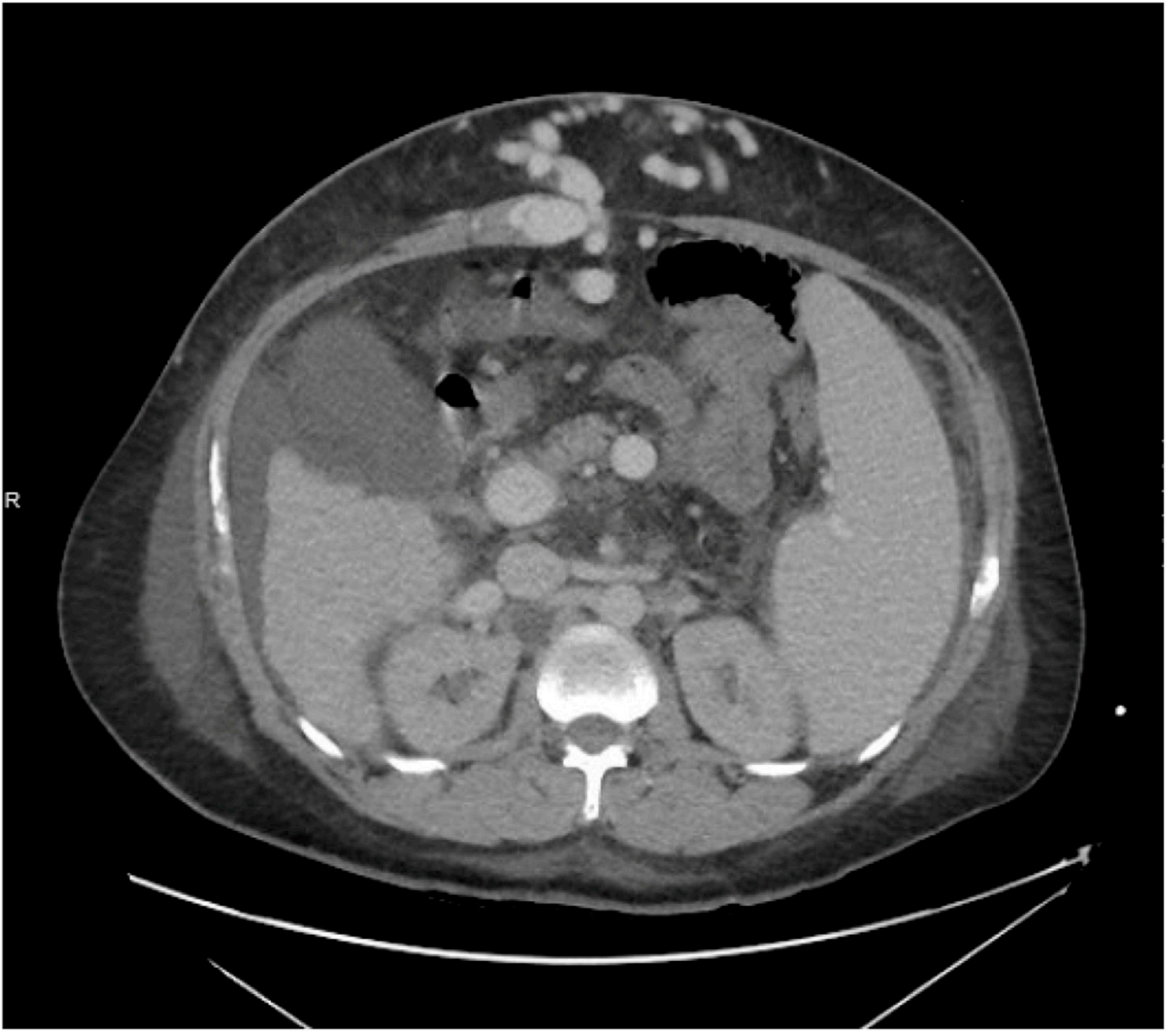
Example of a CT scan showing varices within the hernia sac and splenomegaly (contraindications to robotic hernia repair).

All patients underwent pre-operative cross-sectional imaging. Patients with significant abdominal wall varices precluding safe minimally-invasive abdominal access, or large varices within the hernia sac (E.g., Caput Medusae, Figure 1), were not considered candidates for robot-assisted repair.

The robotic platform used was the Da Vinci Xi. Two 8 mm trocars and one 12 mm trocar were used with lateral placement (lateral to the linea semilunaris). First, the spleen was assessed and if there was no splenomegaly, a Veress needle approach was used at Palmer’s point. An 8 mm optical entry was then performed using a 5 mm Stryker camera at the superior trocar site. If significant splenomegaly precluded Veress needle entry at Palmer’s point or if ascites were present, we proceeded with direct optical entry. The second 8 mm trocar, used for the robotic camera, was then placed inferior to the initial trocar at a distance of at least 8–12 cm (approximately one fist length) away. The 12 mm trocar, used for needle and mesh insertion, was again placed at least 8–12 cm inferior to the second trocar to allow for adequate movement of the robotic arms. Care was taken to avoid the inferior epigastric vessels with the inferior trocar placement. Ports were placed laterally along the linea semilunaris so that the skin and fascial incisions were offset for each port site to reduce post-operative ascitic leakage. The laterality of the ports was determined by the laterality of the ventral hernia. If midline, either side is viable. If off midline, the side that gave the most working distance was chosen (i.e., hernias slightly right of midline had ports placed on the left). Adhesions were taken down using a vessel sealing device. A #1 non-absorbable barbed suture was used for primary defect closure. Primary defect closure was performed in all cases, either as primary closure alone or with subsequent mesh placement. In all cases where mesh was used, an 11 × 11 cm Ventralight ST mesh with an echo system was used. The position of the mesh was in the intraperitoneal onlay mesh (IPOM) position. The mesh was secured using a 2–0 absorbable barbed suture. No mesh was used in patients with large volume ascites (defined as ascites requiring serial paracentesis) due to the risk of mesh infection in both spontaneous bacterial peritonitis and secondary bacterial peritonitis with serial paracentesis. No drains were left at the end of the case to reduce fluid shifts in the perioperative period. All port sites (including 8 mm port sites) were closed with a Carter Thomason suture passer. All other steps followed the standard robotic-assisted umbilical hernia repair.

## Results

### Demographics

Seven patients (five with umbilical hernias, and two with ventral incisional hernias) were included (Table 1). Both ventral incisional hernias were midline in the umbilical region (M3), recurrent, and 2 cm in width (W1) according to the European Hernia Society classification system for incisional abdominal wall hernias [7]. The median follow-up was 173 days. The mean age was 57 years old (range 49–62) and the median MELD was 17 (IQR 14–22). The majority of patients (6 of 7) had ascites at the time of surgery and the majority of patients (5 of 7) had previous hernia-related admissions. Three required a paracentesis to manage large-volume ascites within the previous 6 months. Of the patients who did not require a paracentesis, all were on spironolactone and furosemide for ascite management. One patient had TIPS and one patient had a history of portal vein thrombosis. The average hernia size was 7 cm^2^ (SD 4.4 cm^2^). Three patients required urgent surgery.

**TABLE 1:** Baseline Characteristics and outcomes of robotic ventral and umbilical hernia repair in patients with cirrhosis.

**Characteristics**	Pre-transplant (n = 7)
Mean Age, years	57 (SD = 5.10)
Gender = Male	6
Race/Ethnicity Hispanic/Latino White	3 4
Emergent/Elective Surgery Elective Emergent	4 3
Etiology of Liver Disease MASH/ETOH HH ETOH	1 1 5
Median MELD Score	17 (IQR 14–22)
Dialysis = Yes	2
Hernia Type Umbilical Ventral incisional	5 2
Preoperative Medication Spironolactone Furosemide Lactulose	5 5 5
Ascites Present	6
Paracentesis within the previous 6 months	3
History of Portal Venous Thrombosis	1
Previous TIPS	1
Transplant candidate Yes, SLK Yes, LTX Not listed	2 2 3
Hernia Dimensions, cm (length x width)	2 × 2, 2 × 2, 2 × 2, 3 × 2, 3 × 2, 3 × 3, 4 × 4
Mean Hernia Size, cm^2^	7 (SD = 4.4)
At Least One previous Admission for Hernia	5
Median Admission for Hernia	1 (Range 0–4)
Surgery Mean robotic docking time, min Mean estimated blood loss, cc Total complications Mean length of stay, days Readmission Drains placed at the time of surgery Post-operative leaking ascites from incisions Hernia Recurrence	55.1 (SD = 26.7) 36.9 (SD = 72.4) 2 2.57 (SD = 1.40) 0 0 0 0

ETOH, Alcoholic Cirrhosis; HH, Hereditary Hemochromatosis; LTX, Liver Transplant; MASH, Metabolic Dysfunction-Associated Steatohepatitis; MELD, Model for End-Stage Liver Disease; SLK, Simultaneous Liver-Kidney Transplant; TIPS, Transjugular Intrahepatic Portosystemic Shunt.

### Operative Characteristics and Outcomes

Mean robotic docking time was 55 min (SD 26.7), estimated blood loss (EBL) was 36.9cc (SD 72.37) and mean length of stay was 2.57 days (SD 1.4). Mesh was used in 4 patients with medically managed ascites. There were 2 Clavien-Dindo grade 1–2 complications: one superficial skin infection and one case of urinary retention. There were no Clavien-Dindo grade 3 or higher complications. No patients had post-operative ascitic leakage. No patients had recurrence.

### Urgent Indications

Three patients underwent urgent repair. The first was a patient who was listed for a liver-kidney transplant with recurrent small bowel obstructions on five occasions leading to admission for incarceration each time causing decompensation. The second patient was listed for a liver-kidney transplant with spontaneous leakage of ascites from her umbilical hernia associated with an umbilical ulcer. The third patient had a recurrence of a non-reducible ventral hernia containing fat with severe pain requiring ongoing intravenous pain medication.

## Discussion

This study suggests the safety and efficacy of robotic-assisted umbilical hernia repair in a series of 7 patients with cirrhosis, 6 with refractory ascites and 3 who underwent surgery under urgent conditions. There is a paucity of data on this patient population and robotic hernia repair is scarce, so we believe this is the first series to be published.

The majority of patients with hernias in the setting of cirrhosis and refractory ascites are not being operated on despite significant need. While the optimisation of ascites or repair at transplantation are attractive options, many patients with no imminent access to liver transplants fail attempts to eradicate ascites, especially at centres where average waiting times exceed 1 year. When relegated to “conservative management,” these patients experience recurrent complications such as small bowel obstruction, pain, flood syndrome, and even risk of death each time they have a complication that causes their liver disease to decompensate acutely [6]. Even if decompensations are well managed, these hernias can be the driver for costly emergency room visits and admissions. Despite significant costs, these patients are often not offered surgery for fear of additional complications [8].

If repaired, the majority of these patients receive open repairs, which risk ascitic leakage through the wound and the repair itself and require drain placement and ongoing drain management [9, 10]. Debate exists on the usage of robotic-assisted surgery for other surgeries (e.g., hepatectomy) in patients with cirrhosis, but none has specifically explored its application for hernias [11, 12]. Guidelines have suggested using an open repair in patients with compromised liver function with low quality of evidence [13]. However, several studies have demonstrated the benefit of a minimally invasive repair over an open approach [13–15]. Validated risk calculators specifically in cirrhosis have shown favourable mortality and decompensation rates with minimally invasive surgery (MIS) compared to the open approach [16]. An MIS approach has also been shown to be associated with fewer wound-related complications and a shorter length of stay [15]. One additional point worth articulating is that many of these patients are either transplant candidates or have the potential to be. In these individuals who are likely to require a large incision later on, performing an open repair may increase adhesions and the complexity of the transplant.

A previously suggested exception to MIS repair may be patients with ascites, where laparoscopic surgery has been associated with greater complications in limited data sets [15]. Similar to robotic repair, laparoscopic repair allows for offset, minimally-invasive incisions to lower the risk of leaking from incisions [10, 14, 17]. However, we still advocate instead for robotic repair for several reasons. Laparoscopic repair involves ergonomics that make it more challenging to perform a technically sound primary hernia repair, where the placement and angle of each suture can have a large impact. These intuitions are supported by previous studies suggesting that there is a trend towards increasing robotic surgery in urgent general surgery cases with a lower conversion to open rate and shorter length of stay compared to laparoscopic approaches [18]. While previous studies have suggested that MIS repair is better than open repair in patients with cirrhosis with MELDs above 9 in general, laparoscopic repair has been associated with increased systemic complications and mortality specifically in those with ascites. Thus, achieving MIS closure (with its lower wound complication rates and shorter length of stay) in the setting of ascites may be an indication for robotic repair. Our initial experience shows that robotic repair is feasible with a low complication profile at short-term follow-up. If confirmed in a larger series of patients with cirrhosis undergoing robotic hernia repair, we believe that the cost of the robotic usage would be offset by the quality of the repair and reduced rates of readmissions and complications. We acknowledge that a small ventral hernia defect may be primarily closed laparoscopically by a subset of experienced surgeons. In contrast, robotic primary hernia closure, due to the strength of the robotic arm, articulating instruments and three-dimensional visualisation, can be achieved by the majority if not all surgeons with relative confidence in our experience. Thus, the robotic approach allows for a sound primary hernia repair as the strength of the robotic arms and the range of motion allow for the repair to be completely secured (i.e., with high quality, strong fascial bites and with primary closure prior to any possible mesh placement) as one would do in an open approach. In laparoscopic repair, primary closure is not always routinely performed despite the likely benefit, and as discussed previously it is likely to be more technically challenging [19–21]. In those with minimal ascites, it may be reasonable to consider mesh repair alone (which could be done laparoscopically or robotically) with the risk of recurrence if the patient goes on to develop ascites [13].

The majority of our repairs were closed primarily. While sutured repair with non-absorbable sutures has been reported to result in a high recurrence rate of 15%, even at a short-term 6-month follow-up, there were no recurrences in our cohort with a median follow-up of 6 months [13]. In our experience, the laxity of the abdominal wall and the weakened muscles in the end-stage liver disease population mean that small umbilical hernia defects are rarely closed under tension even when closed primarily. These advantageous factors protect against recurrence in this population, as long as a repair is undertaken. When the mesh was placed, we chose the IPOM location. Guidelines have suggested open repair with onlay or preperitoneal mesh placement for this patient population with low quality of evidence [13]. While the preperitoneal, retrorectus or onlay locations are preferred for standard hernia repair, our decision to use the IPOM location reflects the fact that this is a different category of patient in our experience. Abdominal wall varices and portal-systemic shunts are significant even in patients with compensated cirrhosis. While large varices are visible on cross-sectional imaging, all of these patients have portal hypertension and recanalised portosystemic shunting through the abdominal wall, which may not be readily visible on CT and still increase the risk of intraoperative bleeding, post-operative haematoma around the mesh, and subsequent risk of infection. For these reasons, we advise against preperitoneal/retrorectus dissection. Thus, the overriding principle of our repairs is to perform the repair without major morbidity. Bleeding and haematoma leading to infection are certainly possible and would be much more likely in patients with an INR greater than 2 and a preperitoneal or retrorectus dissection. With respect to adhesions, the 11 × 11 cm mesh in the IPOM location certainly carries a risk of adhesions and a more difficult subsequent liver transplant operation [22]. However, in our cohort, these were generally umbilical hernias and the liver transplant incision is in the upper abdomen, so the risk of the mesh interfering with the subsequent transplant is lower. Additionally, we believe that the risk of an untreated hernia, and the risk of haematoma in the preperitoneal location outweigh the risk of adhesions with the IPOM. However, longer-term follow-up and further studies are needed to better evaluate these competing concerns.

In this report, we offered a strategy to give this underserved population an opportunity at surgery. While expensive upfront, robotic surgery can offer this marginalised population an opportunity at a better quality of life, taking these patients out of a cycle of decompensation and readmission, potentially helping them become or maintain transplant candidacy, which is life extending. From a technical point of view, we advocate 1) reviewing the CT scan to confirm that the entry area and hernial sac have no varices, 2) assessing for significant splenomegaly which may also affect entry trocar placement, 3) offsetting skin and fascial incisions to reduce the risk of leakage, 4) draining only enough ascites to see the working area, and 5) ensuring that the anesthesia team adequately replenishes ascitic losses in the operating room. These steps should serve as a baseline framework for minimising surgical risk and risk of decompensation for those undergoing robotic repair. All of our cases were performed at a major liver transplant centre. We also suggest that these repairs should be performed at or in coordination with a transplant centre, so that the patients have access to a transplant or additional expertise were they to decompensate.

It remains important to highlight that there are cases where we believe robotic-assisted surgery is contraindicated, specifically in patients with large varices herniating into the umbilical sac. Previous reports of robotic-assisted surgery in patients with decompensated cirrhosis have also emphasised the special consideration that must be given to trocar placement [23]. As a result, we believe that a contrast-enhanced CT scan including the venous phase is mandatory prior to surgery. In cases where contrast-enhanced CT is contraindicated (e.g., in patients with compromised renal function), we recommend non-contrast CT as an initial screening test and if there is suspicion of abdominal wall varices, MRI with a gadolinium-based contrast agent may be performed for better characterisation [24]. The risk of bleeding, and the risk of altering the mesenteric drainage by ligating a dominant varix during the hernia repair must be considered on a case-by-case basis. Generally, large varices should not be ligated, if possible, as this results in an abrupt increase in portal hypertension.

This study is limited by the small number of patients in this case series which limits the generalisability of the results. Our case series also reflects a heterogeneous population, patients with and without ascites, various levels of ascites, candidates, and non-candidates for transplantation and urgent versus elective surgery. Nevertheless, we believe that the description of these results in this underserved population with limited treatment options is warranted to stimulate further study. Even the largest societal guidelines have a self-acknowledged weak body of evidence supporting them [13]. Thus we believe that our case series provides valuable additional discussion in a relatively data-sparse area. An additional limitation is selection bias. No patients were refused surgery at our centre during the study period, but patients from referral hospitals with decompensated cirrhosis and hernias may not have been referred. Our centre is also a quaternary liver transplant centre with access to robotic-assisted surgery for both elective and urgent conditions. While this is something that other centres may be considering and may be growing in use [18], we acknowledge that there are limitations to the immediate widespread application of this technique. Our study also only involves mesh placement in the IPOM location for reasons previously discussed. Studies investigating mesh placement in other positions would better clarify the risk/benefit of different mesh positions in this patient population. Finally, the median follow-up was limited to approximately 6 months which is too short to fully assess the risk of recurrence. While our series suggests that robotic-assisted repair is feasible in the short term, additional studies are required to assess long-term outcomes.

In summary, we believe that this series demonstrates that robot-assisted repair can be offered to selected patients with cirrhosis even if they have refractory ascites when the hernia is symptomatic or when a transplant is not on the horizon. While this is a limited series, this establishes a framework for approaching these challenging cases and suggests that further refinement may be possible. As technical expertise in robotic-assisted surgery grows, robotic-assisted ventral hernia repair in patients with cirrhosis with refractory ascites is a promising frontier to provide access to a needed intervention in an underserved population.

## Data Availability

The original contributions presented in the study are included in the article/supplementary material, further inquiries can be directed to the corresponding author.

## References

[1] Pipek LZ Cortez VS Taba JV Suzuki MO do Nascimento FS de Mattos VC Cirrhosis and Hernia Repair in a Cohort of 6352 Patients in a Tertiary Hospital: Risk Assessment and Survival Analysis. Medicine (2022) 101(45):e31506. 10.1097/md.0000000000031506 36397364 PMC9666189

[2] Strainiene S Peciulyte M Strainys T Stundiene I Savlan I Liakina V Management of Flood Syndrome: What Can We Do Better? World J Gastroenterol (2021) 27(32):5297–305. 10.3748/wjg.v27.i32.5297 34539133 PMC8409160

[3] Coelho JC Claus CM Campos AC Costa MA Blum C. Umbilical Hernia in Patients with Liver Cirrhosis: A Surgical Challenge. World J Gastrointest Surg (2016) 8(7):476–82. 10.4240/wjgs.v8.i7.476 27462389 PMC4942747

[4] Salamone G Licari L Guercio G Campanella S Falco N Scerrino G The Abdominal Wall Hernia in Cirrhotic Patients: A Historical Challenge. World J Emerg Surg (2018) 13:35. 10.1186/s13017-018-0196-z 30065783 PMC6064098

[5] Bronswijk M Jaekers J Vanella G Struyve M Miserez M van der Merwe S. Umbilical Hernia Repair in Patients with Cirrhosis: Who, when and How to Treat. Hernia (2022) 26(6):1447–57. 10.1007/s10029-022-02617-7 35507128

[6] Pinheiro RS Andraus W Waisberg DR Nacif LS Ducatti L Rocha-Santos V Abdominal Hernias in Cirrhotic Patients: Surgery or Conservative Treatment? Results of a Prospective Cohort Study in a High Volume Center: Cohort Study. Ann Med Surg (Lond) (2020) 49:9–13. 10.1016/j.amsu.2019.11.009 31853365 PMC6911966

[7] Muysoms FE Miserez M Berrevoet F Campanelli G Champault GG Chelala E Classification of Primary and Incisional Abdominal Wall Hernias. Hernia (2009) 13(4):407–14. 10.1007/s10029-009-0518-x 19495920 PMC2719726

[8] Mahmud N Goldberg DS Abu-Gazala S Lewis JD Kaplan DE. Modeling Optimal Clinical Thresholds for Elective Abdominal Hernia Repair in Patients with Cirrhosis. JAMA Netw Open (2022) 5(9):e2231601. 10.1001/jamanetworkopen.2022.31601 36098965 PMC9471978

[9] Kim SW Kim MA Chang Y Lee HY Yoon JS Lee YB Prognosis of Surgical Hernia Repair in Cirrhotic Patients with Refractory Ascites. Hernia (2020) 24(3):481–8. 10.1007/s10029-019-02043-2 31512088

[10] Cassidy DE Shao Z Howard R Englesbe MJ Dimick JB Telem DA Variability in Surgical Approaches to Hernias in Patients with Ascites. Surg Endosc (2024) 38(2):735–41. 10.1007/s00464-023-10598-6 38049668 PMC11488489

[11] Murtha-Lemekhova A Fuchs J Hoffmann K. Innovation for the Sake of Innovation? How Does Robotic Hepatectomy Compare to Laparoscopic or Open Resection for HCC-A Systematic Review and Meta-Analysis. Cancers (Basel) (2022) 14(14):3359. 10.3390/cancers14143359 35884420 PMC9318519

[12] Di Benedetto F Magistri P Di Sandro S Sposito C Oberkofler C Brandon E Safety and Efficacy of Robotic vs Open Liver Resection for Hepatocellular Carcinoma. JAMA Surg (2023) 158(1):46–54. 10.1001/jamasurg.2022.5697 36416833 PMC9685546

[13] Henriksen NA Kaufmann R Simons MP Berrevoet F East B Fischer J EHS and AHS Guidelines for Treatment of Primary Ventral Hernias in Rare Locations or Special Circumstances. BJS Open (2020) 4(2):342–53. 10.1002/bjs5.50252 32207571 PMC7093793

[14] Salgado-Garza G Patel RK Gilbert EW Sheppard BC Worth PJ. Minimally Invasive Umbilical Hernia Repair Is Safe for Patients with Liver Dysfunction: A Propensity-Score-Matched Analysis of Approach and Outcomes Using ACS-NSQIP. Surgery (2024) 176(3):769–74. 10.1016/j.surg.2024.04.036 38862279

[15] Juo YY Skancke M Holzmacher J Amdur RL Lin PP Vaziri K. Laparoscopic versus Open Ventral Hernia Repair in Patients with Chronic Liver Disease. Surg Endosc (2017) 31(2):769–77. 10.1007/s00464-016-5031-6 27334967

[16] Mahmud N Fricker Z Panchal S Lewis JD Goldberg DS Kaplan DE. External Validation of the VOCAL-Penn Cirrhosis Surgical Risk Score in 2 Large, Independent Health Systems. Liver Transpl (2021) 27(7):961–70. 10.1002/lt.26060 33788365 PMC8283779

[17] Biggins SW Angeli P Garcia-Tsao G Ginès P Ling SC Nadim MK Diagnosis, Evaluation, and Management of Ascites, Spontaneous Bacterial Peritonitis and Hepatorenal Syndrome: 2021 Practice Guidance by the American Association for the Study of Liver Diseases. Hepatology (2021) 74(2):1014–48. 10.1002/hep.31884 33942342

[18] Lunardi N Abou-Zamzam A Florecki KL Chidambaram S Shih IF Kent AJ Robotic Technology in Emergency General Surgery Cases in the Era of Minimally Invasive Surgery. JAMA Surg (2024) 159(5):493–9. 10.1001/jamasurg.2024.0016 38446451 PMC10918578

[19] Bernardi K Olavarria OA Liang MK. Primary Fascial Closure during Minimally Invasive Ventral Hernia Repair. JAMA Surg (2020) 155(3):256–7. 10.1001/jamasurg.2019.5088 31877208

[20] Nguyen DH Nguyen MT Askenasy EP Kao LS Liang MK. Primary Fascial Closure with Laparoscopic Ventral Hernia Repair: Systematic Review. World J Surg (2014) 38(12):3097–104. 10.1007/s00268-014-2722-9 25145817

[21] Bernardi K Olavarria OA Holihan JL Kao LS Ko TC Roth JS Primary Fascial Closure during Laparoscopic Ventral Hernia Repair Improves Patient Quality of Life: A Multicenter, Blinded Randomized Controlled Trial. Ann Surg (2020) 271(3):434–9. 10.1097/sla.0000000000003505 31365365

[22] Jamry A Jałyński M Piskorz L Brocki M. Assessment of Adhesion Formation after Laparoscopic Intraperitoneal Implantation of Dynamesh IPOM Mesh. Arch Med Sci (2013) 9(3):487–92. 10.5114/aoms.2013.35345 23847671 PMC3701981

[23] Li MJ Sprinkles KR Elfedaly M Soliman B. A Case of Robotic Cholecystectomy in a Patient with Decompensated Cirrhosis and Portal Hypertension. Cureus (2024) 16(8):e68315. 10.7759/cureus.68315 39350858 PMC11441718

[24] Woolen SA Shankar PR Gagnier JJ MacEachern MP Singer L Davenport MS. Risk of Nephrogenic Systemic Fibrosis in Patients with Stage 4 or 5 Chronic Kidney Disease Receiving a Group II Gadolinium-Based Contrast Agent: A Systematic Review and Meta-Analysis. JAMA Intern Med (2020) 180(2):223–30. 10.1001/jamainternmed.2019.5284 31816007 PMC6902198

